# The Piwi‐piRNA pathway: road to immortality

**DOI:** 10.1111/acel.12630

**Published:** 2017-06-27

**Authors:** Ádám Sturm, András Perczel, Zoltán Ivics, Tibor Vellai

**Affiliations:** ^1^ Department of Genetics Eötvös Loránd University Budapest Hungary; ^2^ MTA‐ELTE Protein Modelling Research Group Institute of Chemistry Eötvös Loránd University Budapest Hungary; ^3^ Division of Medical Biotechnology Paul Ehrlich Institute 63225 Langen Germany; ^4^ MTA‐ELTE Genetics Research Group Budapest Hungary

**Keywords:** cancer, CRISPR‐Cas, genomic instability, mechanisms of aging, mortality rate, nonaging cells, the Piwi‐piRNA pathway, transposable element

## Abstract

Despite its medical, social, and economic significance, understanding what primarily causes aging, *that is*, the mechanisms of the aging process, remains a fundamental and fascinating problem in biology. Accumulating evidence indicates that a small RNA‐based gene regulatory machinery, the Piwi‐piRNA pathway, represents a shared feature of nonaging (potentially immortal) biological systems, including the germline, somatic cancer stem cells, and certain ‘lower’ eukaryotic organisms like the planarian flatworm and freshwater hydra. The pathway primarily functions to repress the activity of mobile genetic elements, also called transposable elements (TEs) or ‘jumping genes’, which are capable of moving from one genomic locus to another, thereby causing insertional mutations. TEs become increasingly active and multiply in the genomes of somatic cells as the organism ages. These characteristics of TEs highlight their decisive mutagenic role in the progressive disintegration of genetic information, a molecular hallmark associated with aging. Hence, TE‐mediated genomic instability may substantially contribute to the aging process.

Intense investigation in aging research has led to the identification of over five hundred evolutionarily conserved genes, the mutational or RNA interference‐mediated inactivation of which slows down the rate of the aging process in divergent eukaryotic species (Kenyon, 2010). While many of these genetic interventions can significantly promote longevity, they are unable to halt aging. Even mutant animals with extreme longevity continue to age, albeit at a diminished rate when contrasted with their corresponding controls, and eventually die. One of the most striking examples is represented by a gonad‐ablated *daf‐2* mutant *Caenorhabditis elegans* strain that is simultaneously defective for germline activity and insulin/IGF signaling (*daf‐2* encodes a receptor tyrosine kinase that is the *C. elegans* insulin/insulin‐like growth factor receptor ortholog). These nematodes live approximately four times as long as normal (Arantes‐Oliveira *et al*., 2003). In human terms, this lifespan extension would correspond to ~350 years. Longevity genes identified so far thus appear to regulate the rate at which cells age, or suppress the cause of a pathology limiting lifespan to some extent, but the exact mechanisms by which they influence lifespan remain largely unknown. Indeed, understanding what primarily causes aging is still generally considered a great challenge in biology, with significant medical, economic, and social implications (Kenyon, 2010; Baudisch & Vaupel, 2012; Gems & Partridge, 2013; López‐Otín *et al*., 2013).

A related problem in aging research is that of the mortality rate, which displays an exponential growth throughout the adult life in numerous animal species, including humans (Baudisch & Vaupel, 2012). In practical terms, the age of an organism correlates exponentially with the organism's risk to acquire a fatal disease and, eventually, to die. As the accumulation of mutations and harmful metabolic factors, such as reactive oxygen species, causing cellular damage, in particular, truncated, misfolded, oxidized, and aggregated proteins that interfere with cellular homeostasis and functions, is known to occur at a nearly constant rate during the lifespan, the causal role of somatic mutations and intracellular metabolism in the aging process remains unresolved. This issue has bred speculations regarding potential genetic or metabolic components that are likely to be generated exponentially, and to primarily contribute to aging (Kirkwood & Proctor, 2003; Kirkwood, 2008).

Triggered by unrepaired mutations, genomic instability is a key feature of aging cells (López‐Otín *et al*., 2013). Nonaging biological systems however show either no or only limited signs of genome disintegration. Such potentially immortal systems involve the germline that genetically interconnects the subsequent generations, somatic cancer stem cells with indefinite proliferation capacity, and certain organisms from some ‘lower’ animal taxa (e.g. *Planaria* and *Cnidaria*), somatic cells of which display stem cell‐like features (Kyriazis, 2014). The term of ‘nonaging cells’ refers to cells constituting a tissue that traces an essentially immortal lineage. Nonaging tissues display an indefinite renewal capacity. In nonaging cells, genome integrity remains largely stable during the lifespan. ‘Aging cells’ refer to cells constituting a tissue that ages—gradually deteriorates—over time. Genomic instability in aging cells progressively increases during adulthood, thereby limiting their capacity to proliferate and survive. A molecular machinery primarily responsible for maintaining the integrity of genetic material is the Piwi‐piRNA (P‐element‐induced wimpy testis in *Drosophila—*Piwi‐interacting noncoding RNA) pathway (Aravin *et al*., 2007). This small RNA‐based gene regulatory system operates predominantly in nonaging cells (Sedivy *et al*., 2013; Ross *et al*., 2014; Sturm *et al*., 2015). The pathway was originally discovered in the *Drosophila* male germline, and established to function in repressing the activity of mobile genetic elements, also called transposable elements (TEs) or ‘jumping genes’. It is also active in various tumorous cell lines (reviewed by Ross *et al*., 2014), implying that nonaging somatic cancer stem cells adopt certain germline‐specific characteristics, *that is,* some extent of soma‐to‐germline transformation, including the activity of the Piwi‐piRNA pathway and an unlimited proliferation capacity. In addition, certain planaria and cnidaria, such as the planarian flatworms and freshwater hydra, respectively, somatically express components of the Piwi‐piRNA pathway, rendering the self‐renewal ability of their somatic cells apparently unlimited (Martinez, 1998; Petralia *et al*., 2014). These organisms can reproduce clonally, that is the progeny can actually ‘regenerate’ from somatic cells of the parental body. Thus, besides the germline, the Piwi‐piRNA pathway is also active in essentially all types of nonaging somatic cells in diverse organisms, including somatic stem cells in sponge, jellyfish, planaria (in this organism, totipotent stem cells are called neoblasts), sea slug, fruit fly (e.g. in *Drosophila,* the germline function of Piwi proteins depends on the somatic cells of the gonad; Cox *et al*., 1998), sea squirt, and mammals (reviewed in Ross *et al*., 2014). In humans, various somatic cancers and hematopoietic stem cells are known for the activity of the Piwi‐piRNA pathway. These somatic cells/tissues exhibit a largely or essentially unlimited proliferation/renewal capacity, and the pathway functions not only in TE silencing but also in various other cellular processes including epigenetic programming, regeneration, and proliferation. It is intriguing that in the postmitotic organism *D. melanogaster* (‘postmitotic’ means that somatic cells no longer proliferate after completing development), somatic tissues and organs expressing Piwi proteins, such as the gonad, brain, salivary gland, and fat body, are prone to form cancer or at least contribute to the reactivation of dormant self‐renewing progenitor—blast—cells (Sousa‐Nunes *et al*., 2011; Ross *et al*., 2014; Jones *et al*., 2016). Piwi proteins in the fly may thereby also accumulate in somatic stem cells.

TEs are capable of moving from one genomic locus to another, frequently generating insertional mutations in functional DNA regions (Malone & Hannon, 2009; Levin & Moran, 2011). These highly repetitive genetic elements are effectively repressed by the activity of the Piwi‐piRNA pathway in nonaging germline and somatic cells. In contrast, aging somatic cells, in which the Piwi‐piRNA pathway is inactive, are susceptible to significant levels of transposition. Although TEs were previously considered inert DNA stretches labeled ‘junk DNA’, accumulating evidence has revealed that many types of TEs become increasingly mobile in the genomes of somatic cells as the organism ages (Kazazian, 2011; Huang *et al*., 2012). In good accordance with these findings, the increasing activity of TEs is linked to the incidence of various age‐associated degenerative pathologies (O'Donnell & Burns, 2010; Li *et al*., 2013; Kreiling *et al*., 2017). The cumulative mutagenic effect of TE‐derived insertions is likely highly significant, partly because TEs constitute a significant portion of eukaryotic genomes (for instance, the human genome contains thousands of copies of active TEs; Huang *et al*., 2012), and partly because novel TE insertions represent a far higher mutagenic load to the cell than mutations generated by chemical or physical mutagens. TE insertions consist of normal, chemically unaltered nucleotides, and therefore cannot be recognized and eliminated by the otherwise effective DNA repair mechanisms. In eukaryotic genomes, the great majority of TEs belong to the class of self‐duplicating retrotransposons, which are mobilized via the so‐called ‘copy‐and‐paste’ replication mechanism; the original TE does not get excised from its donor locus while the novel copy jumps into a different genomic position. Continuous mobilization of such elements gradually increases their own copy number over the adult lifespan (De Cecco *et al*., 2013a). This may cause an exponential mutation rate in the genomes of somatic cells as the organism ages (Sturm *et al*., 2015). If the mobilization of retrotransposons indeed displays an exponential rate in aging somatic cells, the growth dynamics of their copy number could correlate to the mortality pattern of many animal species (Fig. 1).

**Figure 1:**
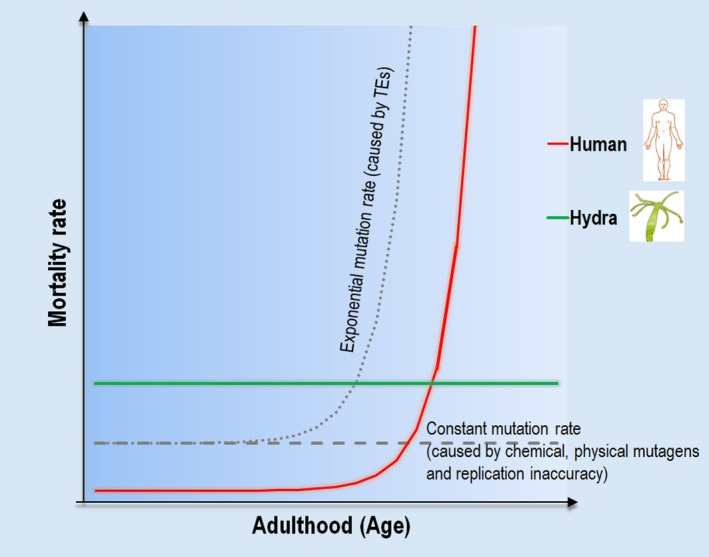
A possible correlation between mortality and mutation rates in humans and hydra. In the potentially immortal freshwater hydra, in which the Piwi‐piRNA pathway is active both somatically and in the germline, inhibiting TE activity essentially in the whole body, a risk of acquiring a fatal disease does not increase with age (the horizontal green line). In this organism, metabolic (e.g. reactive oxygen species), environmental (e.g. high temperatures), and genetic (spontaneous mutations from DNA replication inaccuracy and mutations induced by chemical/physical agents) factors generate cellular damage at a nearly constant rate (the horizontal dashed gray line). Damaged cytoplasmic constituents produced this way can be effectively eliminated by repair/maintenance (cell cleaning) systems. Rarely, when the elimination is unsuccessful, the affected cells become lost, thereby maintaining the functionality of the somatic tissue. In humans, however, in addition to these mutagenic and damaging factors, TEs generate damaged (mutant) intracellular proteins at an increasing rate in somatic cells throughout the lifespan (dotted gray curve). In these cells, the Piwi‐piRNA pathway is largely inactive, which allows self‐replicating TEs to accumulate exponentially. When the level of damages passes a critical threshold, the (saturated) repair/maintenance systems cannot eliminate all of them, leading to a significant amount of cell death. As a consequence, an age‐associated fatal disease can develop, leading to mortality along an exponential rate (red curve). Thus, the exponential mortality rate in humans could be correlated with the exponential mutational rate caused by TEs.

In aging somatic cells, the Piwi‐piRNA pathway is largely inactive. Its TE‐inhibiting function can be somewhat substituted by another small RNA‐guided gene regulatory mechanism, the siRNA (small interfering RNA) pathway, which in various organisms is also capable of silencing TE‐derived mRNAs through processing endogenous double‐stranded RNA structures (Ghildiyal *et al*., 2008). In certain organisms, such as plants that dispense with the Piwi‐piRNA pathway, the siRNA‐mediated silencing system appears to act as the main defense mechanism against the mobilization of TEs in both soma and germline. However, the siRNA pathway functions less effectively than the piRNA pathway in silencing TEs due to three factors: It (i) represses TE transcripts only when they are processed through dsRNS intermediates, (ii) has a reduced capacity to pack silenced TEs into heterochromatin, a tightly packed chromosomal structure (Law & Jacobsen, 2010), and iii) does not involve a piRNA cluster‐like genomic ‘library’ system, which contains a representative copy of each TE family, for more efficient recognition of the corresponding TE transcripts (Aravin *et al*., 2007).

In the absence of active Piwi‐piRNA pathway components, aging somatic cells tend to increasingly lose heterochromatin, which normally maintains TEs under transcriptional repression (Heyn *et al*., 2012; Savva *et al*., 2013; Gorbunova *et al*., 2014; Wood *et al*., 2016). Thus, during adulthood, the gradual release of TEs may generate considerable levels of molecular damage that overwhelm the capacity of the cellular maintenance and DNA repair systems, including autophagy, the ubiquitin–proteasome system, molecular chaperones, and the distinct DNA repair pathways (Fig. 2). The affected cell may become compromised, and then eliminated via cell death to ensure the functional integrity of the somatic tissue. The repair and maintenance (cell cleaning) mechanisms are likely to be equally effective in the soma and germline in eliminating damages produced by metabolic (e.g. reactive oxygen species) and environmental (e.g. heat or UV radiation) factors, as well as those caused by transposition‐independent mutations (i.e. induced by chemical and physical mutagens, or resulting from replication inaccuracy) that occur at a nearly constant rate throughout the lifespan (Fig. 2). In addition to their increasing mobilization during adult life, TEs can inactivate genes that function in the repair and maintenance systems, further contributing to the age‐associated accumulation of cellular damage. Unrepaired cellular damage can frequently cause cell loss. Elevated levels of cell death may then trigger tissue deterioration associated with an age‐related pathology, and, eventually, organismal death. In contrast, the Piwi‐piRNA pathway protects the germline and nonaging somatic cells from TE‐mediated mutagenesis. Occasional mutations generated by chemical and physical mutagens are effectively recognized and eliminated by cellular maintenance and repair mechanisms, and if not, the affected cell is removed from the tissue via cell death (Vellai, 2009; Vellai *et al*., 2009; Vellai & Takács‐Vellai, 2010). Therefore, nonaging cells (tissues) are potentially immortal as their genomes remain largely intact and stable. In other words, the Piwi‐piRNA pathway may play a critical role in cellular immortality, and TEs may represent the primary genetic determinants of aging (Sturm *et al*., 2015) (Fig. 2).

**Figure 2:**
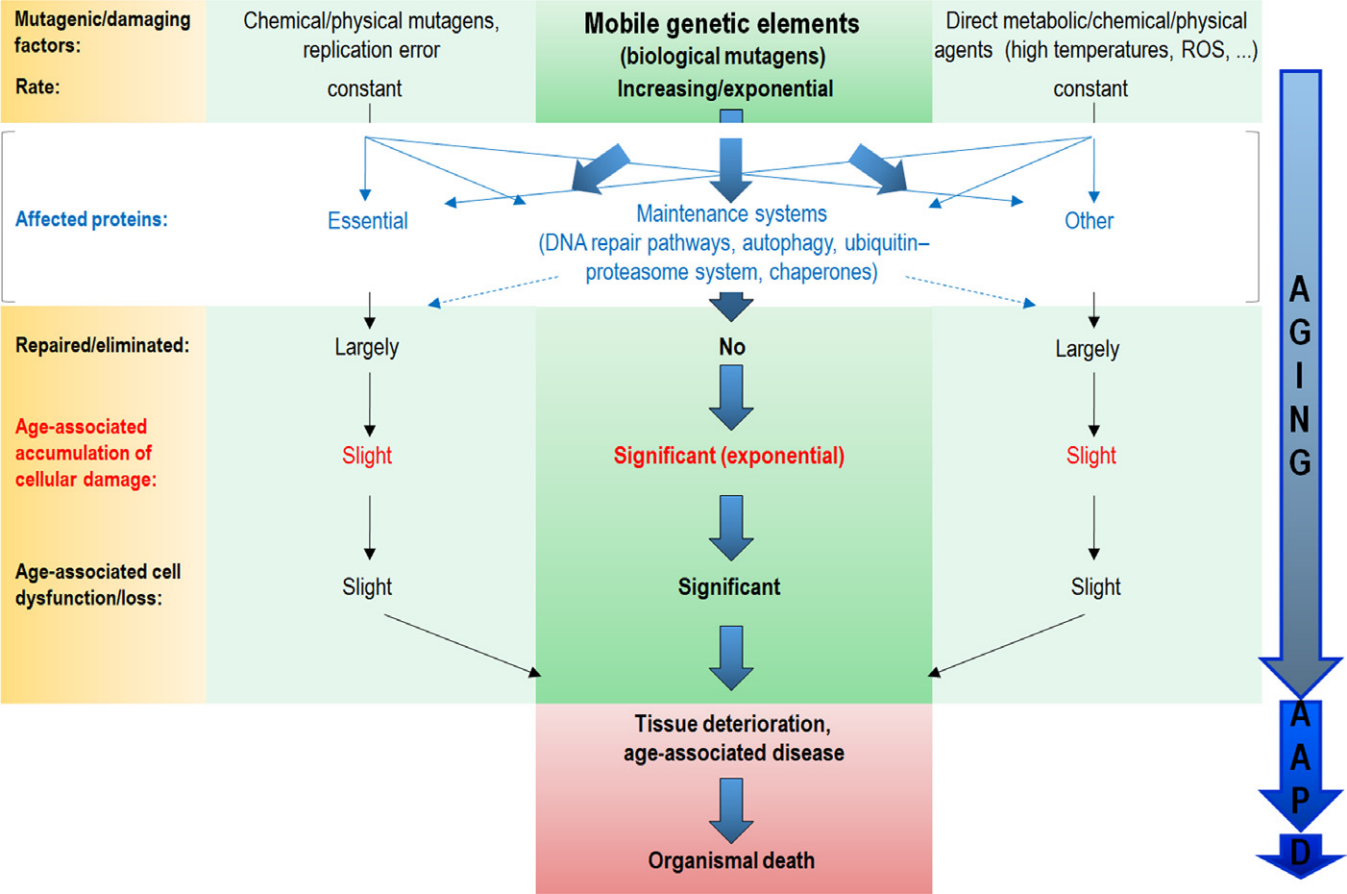
A model of aging driven by transposable element activity. Aging is driven by the progressive, lifelong accumulation of unrepaired cellular damage. Such damages mainly include oxidized, aggregated, or misfolded (nonfunctional) proteins that can act as cellular toxins, thereby compromising cell function and viability. Damage can be produced by injurious exogenous and endogenous factors such as high temperatures and reactive oxygen species, or by mutations generated spontaneously (from replication error) or caused by chemical and physical mutagens. These damages are incurred at a nearly constant level in both soma and germline throughout the adult lifespan, and can be effectively repaired or eliminated at the DNA or protein level by the repair/maintenance systems, including the distinct DNA repair pathways, autophagy (the main mechanism of cellular self‐degradation), the proteasome–ubiquitin system, and molecular chaperones. Occasionally, if repair/degradation fails, the compromised cell is rapidly lost via cell death, thus maintaining the integrity of the tissue. In aging somatic cells, however, in which the Piwi‐piRNA pathway is not active, the mobilization of TEs (biological mutagens) generates additional mutations at an increasing rate throughout the adult lifespan, thereby causing severe genomic instability at advanced ages. The TE‐induced mutations remain unrepaired, and lead to further protein damages that increasingly accumulate in the cytoplasm. Under a critical threshold, TE‐derived damages are also eliminated by the maintenance systems. When the level of TE‐triggered cellular damages passes this threshold, the affected cell initiates a self‐killing program. Massive levels of cell death then cause a fatal age‐associated disease, and, eventually, death. The mobilization of TEs can mutagenize genes that participate in repair and maintenance systems. For example, when a TE jumps into an autophagy‐related gene in an individual somatic genome, the autophagic process becomes compromised in the affected cell. This mechanism can explain why the capacity of the repair and maintenance systems declines in old organisms, which further contributes to the accumulation of cellular damage in this life period. Thus, TE‐caused genomic instability predominantly contributes to the aging process. In other words, TEs may represent the primary genetic determinants of aging. Thick arrows represent significant effects, while thin ones show slight effects. Within the thick arrows at right: AAP, age‐associated pathology; D, death.

Alternatively, the Piwi‐piRNA pathway may have a different, TE‐independent, but as of yet unexplored function to ensure genomic integrity in nonaging cells. For example, the pathway may regulate the transcription of certain key genes via modulating chromatin organization. It is also possible that besides the Piwi‐piRNA system, another molecular mechanism operates in nonaging cells to preserve the stability of their genomes. Such a mechanism however has not yet been identified. Nevertheless, the activity of the Piwi‐piRNA pathway is a shared feature of all nonaging cells identified so far.

As shown recently in *Drosophila*, the mobilization of TEs gradually increases in the brain during aging (Li *et al*., 2013). However, the cause‐or‐consequence context behind this phenomenon remains unresolved. Whether the aging of the animal is a result of growing TE mobilization, or TEs progressively mobilize because the animal ages, is an issue that these observations cannot address. To unequivocally answer this problem one should simultaneously block the members of an active TE family and detect lifespan extension in the treated organisms. Several other indications for the association of TE activity with senescence were reported in the previous years. For example, in yeast, TEs are highly active in aging mother cells, the genetic integrity of which is severely compromised (Patterson *et al*., 2015). In the *Drosophila* fat body, TEs become derepressed in an age‐dependent manner, and their mobilization is accompanied by the deterioration of the organ and elevated levels of DNA damage (Chen *et al*., 2016). Consistent with these results, the expression of TEs in this organism also progressively increases in neurons, and the suppression of age‐associated TE activation promotes longevity (Wood *et al*., 2016). Moreover, studies on mice have shown that TEs are gradually mobilized and multiplied in different tissues over the adult lifespan, most obviously in the brain (De Cecco *et al*., 2013b; Van Meter *et al*., 2014).

When a novel TE‐like sequence invades a eukaryotic genome, for instance following a retroviral infection, a copy of the new DNA stretch may be transferred into a specific genomic locus called the piRNA cluster. This particular part of the host genome actually collects single copies of all TE families, thereby serving as a ‘genomic memory’ or immune pool to distinguish endogenous (‘self’) DNA sequences, which emerged within the lineage, from foreign (‘nonself’) ones, which emerged from outside the lineage. This acquired immunity‐like genetic system is likely to have evolved for genome maintenance by suppressing deleterious TE activity analogous to that of the CRISPR/Cas (clustered regularly interspaced short palindromic repeats/CRISPR‐associated nuclease system) mechanism, which protects bacterial cells from the effect of infective foreign phages or plasmid DNA. For example, when a phage first infects a bacterial species, a copy of its DNA fragment is inserted into the CRISPR array of the host genome as a novel spacer sequence, which then confers to it a resistance against the same viral DNA (Koonin, 2017). As with short segments of spacer DNA within the CRISPR array, piRNA genes code for transcripts that mediate the sequence‐specific recognition and subsequent degradation of the corresponding TE mRNAs by Piwi family proteins.

Why does a large fraction of eukaryotic genomes encode TEs if these repetitive elements are so mutagenic? We hypothesize that TEs may have a dual role in lifespan determination as, besides perturbing the function of somatic cells through harmful insertional mutations they generate, they can also protect somatic tissues from undergoing tumorigenesis. In other words, TEs can be used as a ‘tool’ for increasing longevity as they can delay organismal death through providing a protection against cancer. Compared with nondividing cells, the faster metabolic rate of tumorous cells is associated with elevated transcriptional activity mediated by chromatin opening, which also allows TE mobilization, leading to genome instability and eventually cell loss. TEs therefore may function as a ‘time bomb’, the ‘ticking’ of which is much faster in hyperproliferating cells than in nondividing cells. Indeed, numerous recent studies have shown that cancerous cells display ectopic expression of Piwi proteins and Piwi‐interacting RNAs, and these factors exert transcriptional and posttranscriptional gene regulatory actions (Siddiqi *et al*., 2012; Hashim *et al*., 2014; Ross *et al*., 2014). Immortal HeLa cells also abundantly accumulate Piwi proteins and express piRNAs (Lu *et al*., 2010). Furthermore, the oncogenic transformation of somatic cells induces a functional piRNA pathway (Fagegaltier *et al*., 2016). Consistent with these data, ectopic expression of Piwi proteins in the soma can initiate tumor growth (Janic *et al*., 2010; Siddiqi *et al*., 2012). Thus, genes acting in the Piwi‐piRNA pathway function as proto‐oncogenes, and their products serve as potent tumor markers (Tan *et al*., 2015). Together, these data imply that a nascent tumor can be stabilized when hyperproliferating cancer stem cells stabilize their genetic integrity via TE repression combined with telomere maintenance. Therefore, tumor cells adopt germline‐specific characteristics, including the ectopic expression of Piwi proteins and piRNAs. As a supporting evidence, in *Drosophila*, mutation‐triggered brain tumors are characterized by the ectopic expression of germline‐specific genes including *vasa*,* piwi*,* aubergine,* and *ago3*, many of which code for proteins in the Piwi‐piRNA pathway (Janic *et al*., 2010). In addition, many of the small regulatory RNAs (such as mi‐ and piRNAs) that accumulate at high levels in brain tumors are also highly expressed in the normal ovary. Tumor cells thus reanimate multiple germ cell features (Wu & Ruvkun, 2010).

As the germline is largely free of TE activity due to a functional consequence of the Piwi‐piRNA pathway, this cell type should be more sensitive to tumorigenesis than those lacking the pathway. Indeed, in the nematode *Caenorhabditis elegans,* the only tissue in which a tumor could be induced is the germline (Kirienko *et al*., 2010). In humans, 95% of testicular cancer is likewise germline‐based (Ye & Ulbright, 2012). Another possible reason for the widespread existence of TEs in eukaryotic genomes may stem from evolutionary benefits: by leading to the elimination of old, postreproductive individuals from the populations, TEs may significantly lower intraspecific competition under conditions of limited resources. Alternatively, TEs may serve to increase genetic diversity through mediating genomic rearrangements.

Finally, if further research indeed pinpoints TE‐mediated insertional mutagenesis as the primary genetic determinant of aging, the question of why genetic analysis has failed to reveal this important function of jumping genes for so long will have to be answered. Forward and reverse genetic approaches rely on the phenotypic characterization of single‐gene mutations and knockdowns. However, TEs are generally present in large copy numbers in eukaryotic genomes. As an example, the human genome encodes around 870 000 LINE (long interspersed nuclear element) and 1 560 000 SINE (short interspersed nuclear element) retrotransposons (Lander *et al*., 2001). Although only a fraction of these elements is transpositionally active, it has been estimated that the diploid human genome contains >100 transpositionally active LINE‐1 elements (Brouha *et al*., 2003), thousands of active copies of the *Alu* retrotransposon (Hormozdiari *et al*., 2011) and ~1000 potentially active copies of the composite retrotransposon SVA (Hancks & Kazazian, 2010). Therefore, it appears to be almost impossible to completely inactivate a certain TE family by inducing mutations in each active member of the family. Gene silencing also becomes ineffective over a defined number of paralogous genes targeted for downregulation. In addition, the contribution of a single TE family to the whole lifespan phenotype is likely to be rather moderate and hence difficult to detect as numerous TE families constitute the repetitive fraction of eukaryotic genomes. Considering these facts, to provide direct evidence that the progressive, lifelong mobilization of TEs represents the primary mechanism of aging will certainly not be an easy task.

## Funding

This work was supported by the grants OTKA (Hungarian Scientific Research Fund) NK78012 and MEDinPROT Protein Science Research Synergy Program (provided by the Hungarian Academy of Sciences).

## Conflict of interest

The authors declare no conflict of interest.
